# Pasteurized Colostrum Improves Blood Immunity and Gastrointestinal Microbiota in Dairy Calves from Birth to 180 Days of Age

**DOI:** 10.3390/microorganisms13092089

**Published:** 2025-09-08

**Authors:** Yimin Zhuang, Xuming Dong, Tianyu Chen, Shuai Liu, Jingjun Wang, Jianxin Xiao, Mei Ma, Wei Wang, Mengmeng Li, Shengli Li, Zhijun Cao, Yajing Wang, Jiaying Ma

**Affiliations:** 1 State Key Laboratory of Animal Nutrition, College of Animal Science and Technology, China Agricultural University, Beijing 100193, China; z1164323345@163.com (Y.Z.); 18734793776@139.com (X.D.); chentianyu@cau.edu.cn (T.C.); liushuaicau@cau.edu.cn (S.L.); wangjingjun@cau.edu.cn (J.W.); xiaojianxin@sicau.edu.cn (J.X.); mamei@cau.edu.cn (M.M.); wei.wang@cau.edu.cn (W.W.); limeng2021@cau.edu.cn (M.L.); lishengli@cau.edu.cn (S.L.); caozhijun@cau.edu.cn (Z.C.); 2 Institute of Animal Culture Collection and Application, College of Animal Science and Technology, Yangzhou University, Yangzhou 225009, China; 3 Animal Nutrition Institute, Sichuan Agricultural University, Chengdu 611130, China

**Keywords:** calf, pasteurized colostrum, gastrointestinal microbiota, blood immunity

## Abstract

Pasteurized colostrum has significantly contributed to improving the health and growth of newborn calves by reducing total bacterial count. However, previous research on animal responses to pasteurized colostrum has primarily focused on physiological functioning and production performance, especially during the preweaning period, with limited attention to any postweaning effects from the feeding of pasteurized colostrum at birth. We conducted a comprehensive investigation into the growth, health, blood immunity, and microbiota responses of dairy calves in these two groups from birth to 180 d of age. In this study, a total of 32 healthy female Holstein calves [mean birth weight = 39.8 ± 1.22 kg (mean ± standard deviation)] were selected and divided into two groups (*n* = 16; fed either pasteurized or unpasteurized colostrum at birth). The results demonstrated that calves fed pasteurized colostrum exhibited enhanced growth performance as indicated by higher body weight (BW) and average daily gain (ADG) compared to those fed unpasteurized colostrum (*p* < 0.05). Calves fed pasteurized colostrum displayed higher lymphocyte ratio (W-SCR) and platelet distribution width (PDW), along with lower neutrophil ratio (W-LCR) and neutrophil count (W-LCC) (*p* < 0.05). Additionally, substantial differences were identified in microbial richness and diversity between the pasteurized and unpasteurized colostrum-fed groups (*p* < 0.05). Distinct microbial communities were observed in the ruminal and fecal regions (*p* < 0.05), and we detected shared beneficial microbiota (*Alloprevotella*, *Parabacteroides*, and *unidentified_Prevotellaceae*) and metabolic functions (metabolism of energy, amino acids, and glycan) in both gut regions of the pasteurized group. Furthermore, our study revealed intricate and robust interactions among microbiota, volatile fatty acid (VFA) and blood indicators (|*r*| > 0.5 and *p* < 0.05). In conclusion, the findings in the present experiment suggest that the positive effects from d 0 pasteurized colostrum feeding may be seen up to d 180, including improved growth performance, health, and blood immunity, and these may be attributed to modifications in microbiota development induced by pasteurized colostrum.

## 1. Introduction

Newborn calves possess a limited capacity to mount an effective immune response in the early stages of life. Consequently, they rely heavily on the vertical transmission of immunoglobulin from their mothers’ colostrum to establish robust immunological defenses [1]. Colostrum is known to contain high levels of immunological proteins, primarily immunoglobulin G (IgG), which can adhere to the surface of the gastrointestinal tract. This attachment enables them to neutralize toxins and agglutinate pathogens, playing a crucial role in maintaining the health of newborn calves [2,3,4]. It has been documented that within the first four hours after birth, the small intestinal epithelium of calves efficiently transports immunoglobulin G (IgG) [5]. However, gut closure occurs with halting the absorption of large molecules including IgG approximately 24 h after birth [6]. Consequently, it has been recommended to ensure that newborn calves receive an adequate supply of colostrum as early as possible, which could maximize their serum IgG concentration and strengthen their immune system.

Colostrum also contains a variety of bioactive compounds that support the growth, development, and immune function of preweaned calves. Colostrum comprises a variety of bioactive components that support the growth, development, and immune function of suckling calves. These components include antimicrobial factors such as immunoglobulins, bioactive oligosaccharides, and lactoperoxidase, as well as immune regulators like cytokines (peptides/proteins and maternal leukocytes) and growth factors such as Insulin-like growth factor 1 (IGF-I), Insulin-like growth factor 2 (IGF-II), Epidermal Growth Factor (EGF), transforming growth factor-alpha (TGF-α), transforming growth factor-beta-1 (TGF-β1), transforming growth factor- beta-2 (TGF-β2) [7], and betacellulin [8]. Studies have consistently demonstrated that calves fed colostrum at birth exhibit enhanced growth performance and greater blood immunity not only in the initial weeks of life, but also throughout the preweaning period [9]. Furthermore, the calves that consumed more colostrum (4L vs. 2L) showed a higher milk yield when they reached adulthood [10]. The rumen of preruminants is still in the developmental stage, and the hindgut serves as the primary site for nutrient digestion and absorption, relying on the combined action of the host and microbiota. Recent studies have demonstrated that colostrum can serve as one of the earliest sources of microbiota involved in intestinal colonization, including various beneficial bacteria [11,12].

Of note, it is important to emphasize the need for colostrum sterilization due to the potential presence of foodborne pathogens originating from infected animal excretion or contaminated farm environments, which may lead to animal infections or diarrhea [13]. Pasteurization, a gentle low-temperature heat treatment technology, has been used in some dairy farms to process colostrum [14]. This method enables the preservation of beneficial substances present in colostrum to the greatest extent possible [15]. Numerous studies have demonstrated the effectiveness of pasteurization in eliminating harmful bacteria, such as *Escherichia coli*, from colostrum [16], while having minimal impact on the fat and protein content of the colostrum [17]. To date, numerous studies have examined the impact of pasteurized colostrum on the health and microbiota of preweaning calves. For example, feeding pasteurized colostrum to newborn calves has been shown to reduce morbidity rates and increase serum IgG levels compared to fresh colostrum [18]. Moreover, research has indicated that pasteurized colostrum can influence the gut microbiota of neonatal calves. Feeding pasteurized colostrum has been found to enhance the abundance of beneficial bacteria such as *Bifidobacterium*, which produce volatile fatty acid (VFA) and lactate, while inhibiting the colonization of *E. coli*, a potential cause of extraintestinal infection in neonatal small intestines [19]. Similarly, within the first 12 h of life, pasteurized colostrum has been observed to decrease the abundance of opportunistic pathogenic *E. coli* and *Escherichia Shigella* in the hindgut mucosa [20]. However, the prolonged effects of pasteurized colostrum on the gastrointestinal microbiota of postweaning calves remain unclear. During the early life of ruminants, including mono-gastric animals, the hindgut harbors a relatively simple microbial community that can be easily influenced by factors such as vertical transmission, diet, and the host’s rearing environment [21]. Considering the interactions between microbes and the host, as well as the preferential colonization advantage of bacteria, early establishment of the gut microbiota may lead to long-lasting or even permanent changes in the host that can affect growth performance and immune capacity [22]. Therefore, we hypothesized that feeding pasteurized colostrum could have positive effects on the gut microbiota of calves even after weaning.

The objective of this study was to assess the impact of feeding pasteurized colostrum at birth on the growth performance, blood immunity, and gut microbiota of calves from birth to postweaning (180 d of age). We hypothesized that feeding pasteurized colostrum would enhance calf growth and improve immune function by reshaping the gut microbiota to enrich beneficial bacterial taxa.

## 2. Materials and Methods

### 2.1. Farm Enrollment

This study was conducted in Ningxia Hui Autonomous region of China, and calves from a local farm (Jinyu Haoxing Animal Husbandry Development Co., Ltd., Wuzhong, China) were enrolled in the study from September 2019 to March 2020.

### 2.2. Colostrum Preparation

Colostrum was collected from multiple healthy dams in advance. The collection and processing of colostrum followed a strict protocol to ensure its quality and safety for feeding to calves. Within 30 min after calving, the initial colostrum was obtained. A digital refractometer, PAL-Colostrum (Atago, Tokyo, Japan), was used to detect the brix value of colostrum, and only colostrum with a value greater than 22% would be retained. Then the qualified colostrum was promptly refrigerated at 4 °C. It was imperative to limit refrigeration to a maximum of 24 h before feeding to maintain colostrum freshness. To prepare the colostrum for further analysis, the colostrum of each cow was equally divided into two groups and the colostrum of each group was thoroughly mixed. One group remained untreated, while the other underwent a pasteurization process. The pasteurization method involved heat-treating the colostrum in a commercial batch pasteurizer at a temperature of 60 °C for 60 min, with a strict adherence to a maximum allowable fluctuation of 0.56 °C during the holding phase. Throughout the process, continuous agitation of the colostrum was maintained. Following the heat treatment, the colostrum was carefully cooled to a temperature of 15.6 °C, set by the program of the pasteurizer. A total of 50 mL of untreated colostrum and pasteurized colostrum was collected separately for further analysis of their characteristics, including nutritional composition and immune factor concentrations measured in milligrams per milliliter (mg/mL). As the result, although pasteurization slightly reduced the nutritional composition and immune factors of colostrum, these indicators in both groups were still within the normal standard (Table S1). Both the untreated and pasteurized colostrum samples were subpackaged and stored at −20 °C in sterile safety bags (6 L/bag) to ensure their integrity for the upcoming feeding to the calves involved in the study. When a calf was born, the frozen colostrum was thawed in the pasteurizer on a 40 °C program to feed the calf.

### 2.3. Animals and Experimental Design

At birth, all calves were promptly weighed and separated from their dams. In order to ensure controlled conditions for the experiment, twin calves and those requiring calving assistance (via breech extraction) were excluded. The enrolled calves were then randomly assigned, based on their birth order, to either the pasteurized colostrum (PC) group or the unpasteurized colostrum (UC) group. Because frozen colostrum took 1 to 2 h to thaw, calves were ensured to be administered 4.0 L of pasteurized or unpasteurized colostrum through a nipple bottle within a maximum of 2 h after their birth. Subsequently, a second feeding of 2.0 L of colostrum, either pasteurized or unpasteurized, was administered approximately 6 h after the first feeding.

Once the number of calves in each feeding group (PC and UC) reached a total of 40, we selected 16 healthy female calves (*n* = 16) from each group after excluding calves with calving assistance and twins (8 out of 40 calves). These selected calves exhibited similar birth weights, with an average of 39.8 ± 1.22 kg. Throughout the study, every enrolled calf was housed on d 1 of life in an individual newborn pen that was bedded with dry sawdust. On d 2, calves were transferred to four pens for group feeding (8 calves per pen, 2 pens assigned to each feeding group); the weaning protocol is shown in Table S2. It is worth noting that the age difference between calves within the same pen did not exceed 3 days. The calves remained in these group pens until weaning (d 60).

During the preweaning period, the calves had unrestricted access to water and were provided with calf starter feed (Table S3). Additionally, the calves received three daily feedings of pasteurized milk at specific times (0800 h, 1400 h, and 1800 h), as outlined in Table S2. Following weaning, the calves from both the PC and UC groups were transferred to the heifer barn and grouped together until the completion of the study, which continued until 180 d of life. In this facility, calves had unrestricted access to water, starter feed, and oat hay (Table S3).

### 2.4. Growth Performance Measurements

The body weight (BW) of the calves was measured before morning feeding at 0800 h of specific days including at birth (d 0), and on d 30, 60, 120, 150, and 180 of life, utilizing a calf weighing machine (Honneur Agriculture and Animal Husbandry Technology Co, Ltd., Beijing, China). The weighing machine is a movable weighing platform, which is wrapped in stainless steel on three sides to restrict the movement of calves and ensure the reliability and stability of their weight data. These measurements allowed for the calculation of the average daily gain (ADG) of the calves over the course of the study.

### 2.5. Sampling and Analysis of Blood, Rumen Contents, and Feces

Upon completion of the experiment on d 180, blood samples were collected from the jugular vein of the calves using 5 mL vacuum tubes without anticoagulants. A portion of the whole blood was transferred to 2 mL centrifuge tubes and stored in a refrigerator at 4 °C for subsequent analysis. For microbiota analysis, a subset of six calves was randomly selected from each group on d 180. Rumen fluid samples, totaling 200 mL, were collected from these selected calves using an oropharyngeal tube with an 8 mm diameter. Fecal samples were obtained rectally using sterile palpation sleeve gloves. To ensure sample integrity, the rumen and fecal samples were immediately placed in liquid nitrogen and subsequently stored at −80 °C for further analysis. These samples were subjected to analysis for VFA and microbial DNA extraction to investigate the microbiota composition.

Hematological parameters of calves, including total leukocyte count (WBC), number of red blood cells (RBC), hemoglobin (HGB), hematocrit (HCT), mean corpuscular volume (MCV), mean corpuscular hemoglobin (MCH), mean corpuscular hemoglobin concentration (MCHC), platelet (PLT), lymphocyte ratio (W-SCR), monocyte ratio (W-MCR), neutrophil ratio (W-LCR), total lymphocyte count (W-SCC), monocyte count (W-MCC), neutrophil count (W-LCC), platelet distribution width (PDW), mean platelet volume (MPV), red cell volume distribution width (RDW-SD), red blood cell distribution width (RDW-CV) and large platelet ratio (P-LCR), were analyzed by an automatic veterinary hematology cell counter (K4500, Hisen Meikang Co., Ltd., Tokyo, Japan).

The rumen fluid and fecal samples (collected from six calves per group) were thawed at 4 °C and then centrifuged at 2500× *g* at room temperature; 1 mL of the supernatant per sample was separated and transferred into a 1.5 mL centrifuge tube containing 0.2 mL of metaphosphoric acid solution (25% *w*/*v*). The mixture was centrifuged at 10,000× *g* at 4 °C after placing it in a water bath for 30 min. The collected supernatant was stored at 4 °C for the subsequent analysis. The volatile fatty acid (VFA) concentration was detected using gas chromatography (GC-6800, Beijing Beifen Tianpu instrument Technology, Co., Ltd., Beijing, China).

### 2.6. Diarrhea Score

Calf feces was scored (as described by Diaz et al. [23] and Zou et al. [24]: 1 = firm, well formed (not hard); 2 = soft, pudding-like; 3 = runny, pancake batter; 4 = liquid, splatters, pulpy orange juice) and recorded at 0800 and 1700 h daily until d 60 to determine diarrheal status of each enrolled calf. Diarrhea was identified as any animal presenting a score of 3 or 4. One independent trained observer collected data for fecal scores. Calves with diarrhea were given quality care according to the farm’s standard procedure (for example, supplementation of electrolytes, injection of antibiotics, etc.). Each day of diarrhea per calf was counted as diarrhea once. From the data collected, diarrhea frequency was calculated as follows [25]: diarrhea frequency (%) = (number of diarrhea calves × days of diarrhea)/(total number of calves × days of whole trial) × 100%.

### 2.7. Analysis of Nutrient Composition of Starter

The samples of starter were ground by a grinder (Yunbang, Suifeng Industry and Trade Co., Ltd., Pinghu City, China) to pass through a 1 mm sieve. Feed samples were analyzed for dry matter (DM) [26], crude fat (ether extraction (EE)) [27], crude protein (CP) [28], and ash [28].

### 2.8. DNA Extraction and Sequencing

The microbial DNA from ruminal and fecal samples (collected from six calves per group) was extracted using a DNeasy PowerSoil Kit (Cat. No. 12888, Qiagen, Valencia, CA, USA). In the quality control step of DNA, two fecal samples did not meet the standard for sequencing, and therefore they were excluded from downstream analysis. Total DNA quality was checked using 1% agarose gel electrophoresis and a Thermo NanoDrop 2000 UV microphotometer (Thermo Fisher Scientific, Waltham, MA, USA). The V3–V4 region was amplified using adaptor-linked universal primers (341F: CCTACGGGRSGCAGCAG; 806R GGACTACVVGGGTATCTAATC) [29]. Diluted genomic DNA was used as a template and a high-fidelity enzyme was utilized in the KAPA HiFi Hotstart ReadyMix PCR kit to conduct PCR, which could ensure the accuracy and efficiency of the amplification. The AxyPrep DNA Gel Recovery kit (AXYGEN Inc., Union City, CA, USA) was used to cut the gel and recover the PCR products. To prevent contamination from reagents, we also conducted a negative control for DNA extraction and PCR amplification. No PCR products of the negative controls were detected from agarose gel. Using a Thermo NanoDrop 2000 UV microphotometer and 2% agarose gel electrophoresis, library quality was checked. A Qubit 2.0 Fluorometer (Thermo Fisher Scientific, Waltham, MA, USA) was performed to calculate the library. Amplicon libraries were sequenced using an Illumina Miseq PE250 platform (Realbio Technology Genomics Institute, Shanghai, China).

### 2.9. Statistical Analysis

Calves were used as the experimental unit, and the homogeneity of variances and normality of the data were tested first using the UNIVARIATE procedure of SAS (version 9.2, SAS Institute Inc., Cary, NC, USA). The PROC MIXED statement of SAS was used with time as a repeated measure for body weight (BW) and ADG. The model included the fixed effects of time, treatment, and time × treatment interaction and calves within treatment as a random effect. Degrees of freedom were calculated using the Kenward–Roger approximation option of the MIXED procedure. VFA and blood parameters were analyzed by one-way ANOVA. Diarrhea days and frequency data were analyzed using the Chi-square test. Differences of *p* < 0.05 were considered significant and 0.05 ≤ *p* < 0.10 was considered a tendency. Calf feed intake was only measured during the preweaning period at the group level, as all calves were housed in group housing. After weaning, animals were moved to a heifer barn and housed with other calves, and thus the feed intake could not be reported.

The Quantitative Insight into Microbial Ecology (QIIME v 1.9.1) tool kit was used to process the raw sequences. Operational taxonomic units (OTUs) at the 97% similarity level were clustered and aligned to the SILVA (v132) database after removing the chimeras and singletons by Usearch software (v7.0.1090). RDP (Ribosomal Database Project) database was used to classify high-quality reads [30].

Alpha diversity of the two groups was compared using a two-tailed Wilcoxon signed-rank test. Beta diversity based on Bray–Curtis was visualized as the principal coordinate analysis (PCoA) plot and calculated the difference between groups using an analysis of similarity (ANOSIM). Procrustes analysis was finished using the function “vegdist” in the R “vegan” package (version 4.2.3).

The linear discriminant analysis (LDA) effect size (LEfSe) was used to identify group-related signature bacteria. Heatmaps were used to visualize the results. The criterion for judging significance was LDA > 2 and *p* < 0.05. The network analysis was performed using the SparCC algorithm. Only the correlations with |*r*| > 0.5 and *p* < 0.05 were preserved. The network was visualized using the Gephi V0.9.2 and Cytoscape V3.5.1. The functional prediction was performed using the method of PICRUSt software (http://picrust.github.io/picrust/index.html accessed on 2 September 2025). Based on the Kyoto Encyclopedia of Genes and Genomes (KEGG), functions were identified and classified.

## 3. Results

### 3.1. Growth and Health Performance

In accordance with the data presented in Figure S1, it is evident that calves that were fed pasteurized colostrum showed a higher preweaning dry matter intake (DMI) of starter (741.67 vs. 694.94 g/day per calf). Table 1 exhibited significantly higher body weight (BW) on d 120 (*p* = 0.03) and throughout the entire observation period (*p* < 0.01) in comparison to those calves that were fed unpasteurized colostrum. Additionally, it was observed that calves fed with pasteurized colostrum demonstrated a greater average daily gain (ADG) during the preweaning phase (*p* = 0.04) when compared to those fed unpasteurized colostrum, while no notable differences were observed in postweaning ADG. Notably, the incidence and frequency of diarrhea did not display any significant differences between the two groups, as indicated in Table 2.

### 3.2. Blood Immune Parameters, Rumen, and Fecal Fermentation

The hematological profile of the serum samples, as presented in Table 3, revealed noteworthy findings. In the group of calves fed pasteurized colostrum, it was observed that the W-SCR exhibited significantly higher values (*p* < 0.01), along with an elevated PDW (*p* = 0.02). Conversely, the W-LCR and W-LCC were observed to be lower (*p* < 0.01 and *p* = 0.02, respectively) in comparison to the unpasteurized group. Furthermore, on d 180, no notable differences were observed in rumen and fecal fermentation between the two groups, as indicated in Table 4.

### 3.3. The Microbial Composition, Diversity, and Structure

**Figure 1:**
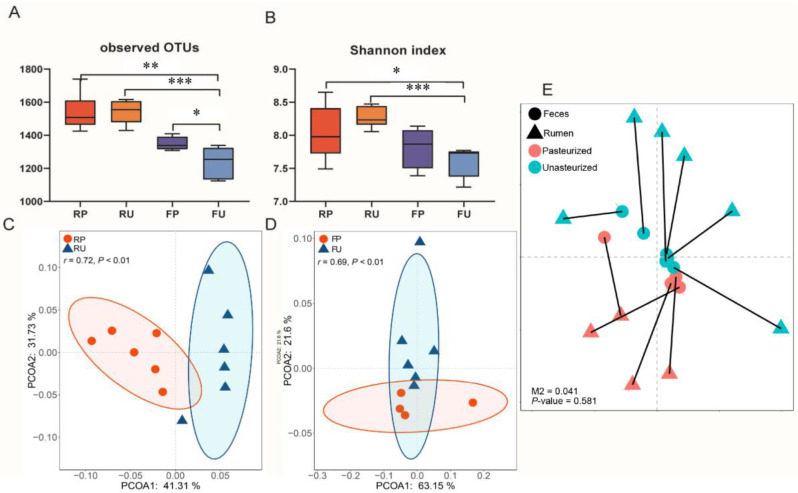
Microbial diversity and structure of the ruminal and fecal microbiota in calves. (**A**,**B**) The changes in alpha diversity of rumen and feces microbial communities based on the Shannon index and Chao1 index. (**C**,**D**) The principal coordinate analysis (PCoA) based on Bray–Curtis with ANOSIM analysis. Each point represents a unique sample. Different colors represent different groups. (**E**) Procrustes analysis of the association between the rumen microbiota and fecal microbiota. The residual, M2, and *p*-values were generated by the ‘protest’ function, with *p* < 0.05 as the significant threshold. RP = rumen samples in the pasteurized colostrum feeding group; RU = rumen samples in the unpasteurized colostrum feeding group; FP = fecal samples in the pasteurized colostrum feeding group; FU = fecal samples in the unpasteurized colostrum feeding group. * *p* < 0.05, ** *p* < 0.01, *** *p* < 0.001.

The rumen and fecal samples in the calves fed with pasteurized colostrum were labeled as the RP and FP groups, respectively. The rumen and fecal samples in the calves fed with unpasteurized colostrum were named the RU and FU groups. Results showed that RP (*p* < 0.01), RU (*p* < 0.01) and FP (*p* = 0.04) had a higher richness (observed OTUs) compared with FU (Figure 1A). Furthermore, the diversity of microbial communities, as measured by the Shannon index, was significantly increased in the RP group (*p* = 0.03) and the RU group (*p* < 0.01) compared to the FU group (Figure 1B). Regarding beta diversity, distinct clustering was observed in the rumen content of both the RP and RU groups (ANOSIM: *r* = 0.702, *p* < 0.01) (Figure 1C). Similarly, a similar pattern of distinct clustering was observed between the FP and FU groups (ANOSIM: *r* = 0.694, *p* < 0.01) (Figure 1D). Furthermore, the consistency between rumen and fecal microbial communities within the same subjects was assessed using Procrustes analysis. However, no significant consistency was found between the rumen and fecal samples in either the pasteurized or unpasteurized colostrum group (M2 = 0.04, *p* = 0.58) (Figure 1E).

**Figure 2:**
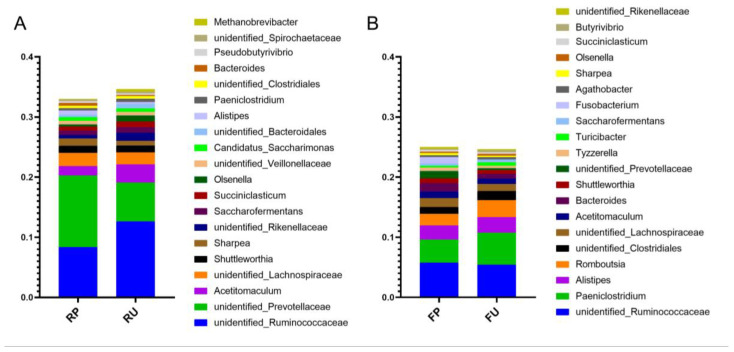
Microbial composition at the genus level in response to pasteurized colostrum feeding. Stacked bar charts demonstrate the abundance of the top 20 common genera in the rumen (**A**) and feces (**B**) of calves, respectively. RP = rumen samples in the pasteurized colostrum feeding group; RU = rumen samples in the unpasteurized colostrum feeding group; FP = fecal samples in the pasteurized colostrum feeding group; FU = fecal samples in the unpasteurized colostrum feeding group.

According to different microbial sample types (rumen or feces), we established four groups to facilitate the comparison and analysis of microbiota. In terms of the microbial composition of rumen content at the phylum level, Bacteroidetes and Firmicutes were dominant in both RP and RU groups. However, Bacteroidetes exhibited a higher abundance in the RP group compared to the RU group, while Firmicutes increased in the RU group (Figure S2A). At the genus level, although *unidentified_Prevotellaceae, unidentified_Ruminococcaceae, Acetitomaculum* and *unidentified_Lachnospiraceae* were dominant in both the RP and RU groups, their abundances exhibited significant differences between the two groups. *unidentified_Prevotellaceae* showed a higher abundance in the RP group compared to the RU group, while *unidentified_Ruminococcaceae* and *Acetitomaculum* displayed the opposite trend (Figure 2A).

Regarding the fecal microbiota composition, the dominant bacterial phyla in FP and FU groups were also *Bacteroidetes* and *Firmicutes.* In the FP group, *Bacteroidetes* exhibited a higher abundance and Firmicutes showed a lower abundance in FP group compared to the FU group (Figure S2B). At the genus level, *Paeniclostridium, unidentified_Ruminococcaceae, Alistipes* and *Romboutsia* were the most abundant bacteria in both the RP and RU groups. However, the abundances of *Paeniclostridium* and *Romboutsia* genera were lower in the FP group compared to the FU group (Figure 2B).

### 3.4. The Identified Signature Bacteria

**Figure 3:**
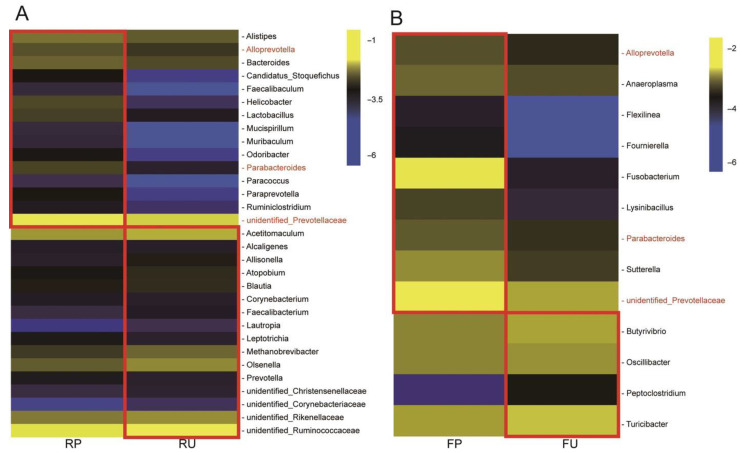
The signature bacteria identified by linear discriminant analysis (LDA) effect size (LEfSe) analysis. The Log-scaled relative abundance heatmap of signature genera screened by LEfSe (LDA > 2, *p* < 0.05) in the rumen (**A**) and fecal (**B**) microbiota, respectively. The genera marked in red color were shared between the RP and FP groups. RP = rumen samples in the pasteurized colostrum feeding group; RU = rumen samples in the unpasteurized colostrum feeding group; FP = fecal samples in the pasteurized colostrum feeding group; FU = fecal samples in the unpasteurized colostrum feeding group.

In order to gain a deeper understanding of the impact of pasteurized colostrum on the gastrointestinal microbial community of the calves, an analysis using LEfSe was conducted. This analysis aimed to identify specific bacteria associated with pasteurization at the genus level in both the rumen and fecal samples (Figure 3). In the rumen content analysis, several genera were identified as signature bacteria in the RP group. These included *Faecalibaculum*, *Mucispirillum*, *unidentified_Paraprevotella*, *Odoribacter*, *Candidatus_Stoquefichus*, *Ruminiclostridium*, *Helicobacter*, *Parabacteroides*, *Lactobacillus*, *Muribaculum*, *Paracoccus*, *Alistipes*, *unidentified_Prevotellaceae*, and *Alloprevotella*. These genera exhibited a higher abundance in the rumen samples of the RP group compared to the RU group. Conversely, the RU group showed signature genera such as *Alcaligenes*, *Methanobrevibacter*, *Atopobium*, *unidentified_Christensenellaceae*, *Acetitomaculum*, *Leptotrichia*, *Blautia*, *Allisonella*, *unidentified_Corynebacteriaceae*, *Lautropia*, *Olsenella*, *unidentified*, *unidentified_Ruminococcaceae*, *Faecalibacterium*, *Prevotella*, *Corynebacterium*, and *unidentified_Rikenellaceae*, which were relatively more abundant compared to the RP group (Figure 3A). In the fecal samples, distinct signature bacteria were observed in the FP and FU groups. The FP group exhibited higher levels of *Flexilinea*, *Fusobacterium*, *unidentified_Prevotellaceae*, *Lysinibacillus*, *Parabacteroides*, *Alloprevotella*, *Sutterella*, *Fournierella*, and *Anaeroplasma* compared to the FU group. On the other hand, the FU group showed signatures such as *Oscillibacter*, *Turicibacter*, *Peptoclostridium*, and *Butyrivibrio* (Figure 3B).

### 3.5. Network Analysis

**Figure 4:**
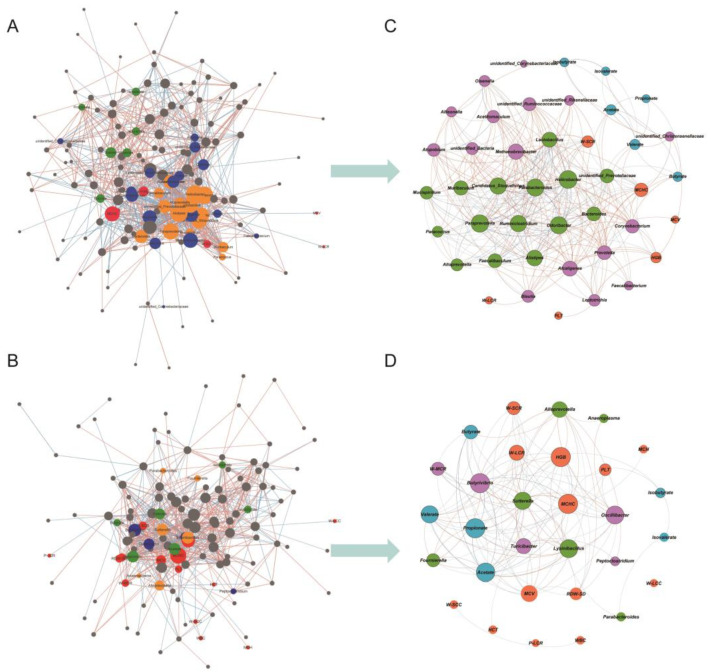
Network interaction analysis. SparCC was used to calculate the correlations among microbiota, blood parameters and VFAs. The global network in the rumen (**A**) and feces (**B**). Different colors of the nodes represent different characteristics (red: blood indicator; green: VFAs; orange: signature bacteria identified by LEfSe in pasteurized colostrum group; deep blue: signature bacteria in unpasteurized colostrum group; grey: other bacteria). The sub-network in the rumen (**C**) and feces (**D**). Different colors of nodes represent different characteristics (orange: blood indicator; light blue: VFAs; green: signature bacteria identified by LEfSe in pasteurized colostrum group; purple: signature bacteria in unpasteurized colostrum group). WBC = total leukocyte count; RBC = number of red blood cells; HGB = hemoglobin; HCT = hematocrit; MCV = mean corpuscular volume; MCH = mean corpuscular hemoglobin; MCHC = mean corpuscular hemoglobin concentration; PLT = platelet; W-SCR = lymphocyte ratio; W-MCR = monocyte ratio; W-LCR = neutrophil ratio; W-SCC = total lymphocyte count; W-MCC = monocyte count; W-LCC = neutrophil count, PDW = platelet distribution width; MPV = mean platelet volume; RDW-SD = red cell volume distribution width; RDW-CV = red blood cell distribution width; P-LCR = large platelet ratio.

The complex interrelationships among key factors, including microbiota, fermentation, and blood parameters, were elucidated through the construction of two networks using the SparCC algorithm (|*R*| > 0.5 and *p* < 0.05). These networks represented the correlations within the rumen content and feces samples, respectively. The global network analysis revealed that the rumen microbiota-related network displayed a higher number of interactions among bacteria, VFAs, and blood parameters compared to the fecal microbiota-related network, with 936 edges and 710 edges, respectively (Figure 4A,B). To highlight the correlation signatures identified by LEfSe within the network, two sub-networks were further constructed. Consistent with the global networks, the rumen microbiota-related sub-network exhibited a greater number of correlations. The signature genera in the RP group served as important junction points, displaying higher connection degrees compared to the RU group. Notably, acetate demonstrated a positive association with *unidentified_Prevotellaceae* and *Prevotella*, while butyrate exhibited a negative association with *Corynebacterium*. The W-SCR emerged as a central feature connecting *Allisonella*, *Paraprevotella*, *Faecalibaculum*, *Mucispirillum*, and other genera within the sub-network (Figure 4C). In the fecal microbiota-related sub-network, butyrate displayed a positive correlation with *Lysinibacillus* and *Alloprevotella*, while valerate was positively associated with *Sutterella*, *Lysinibacillus*, *Alloprevotella*, and *Fournierella*. The W-SCR exhibited negative correlations with *Butyrivibrio* and *Oscillibacter*. Furthermore, the W-LCR showed positive associations with *Butyrivibrio* and *Oscillibacter*, as well as a negative correlation with *Sutterella* (Figure 4D).

### 3.6. Microbial Function Analysis

**Figure 5:**
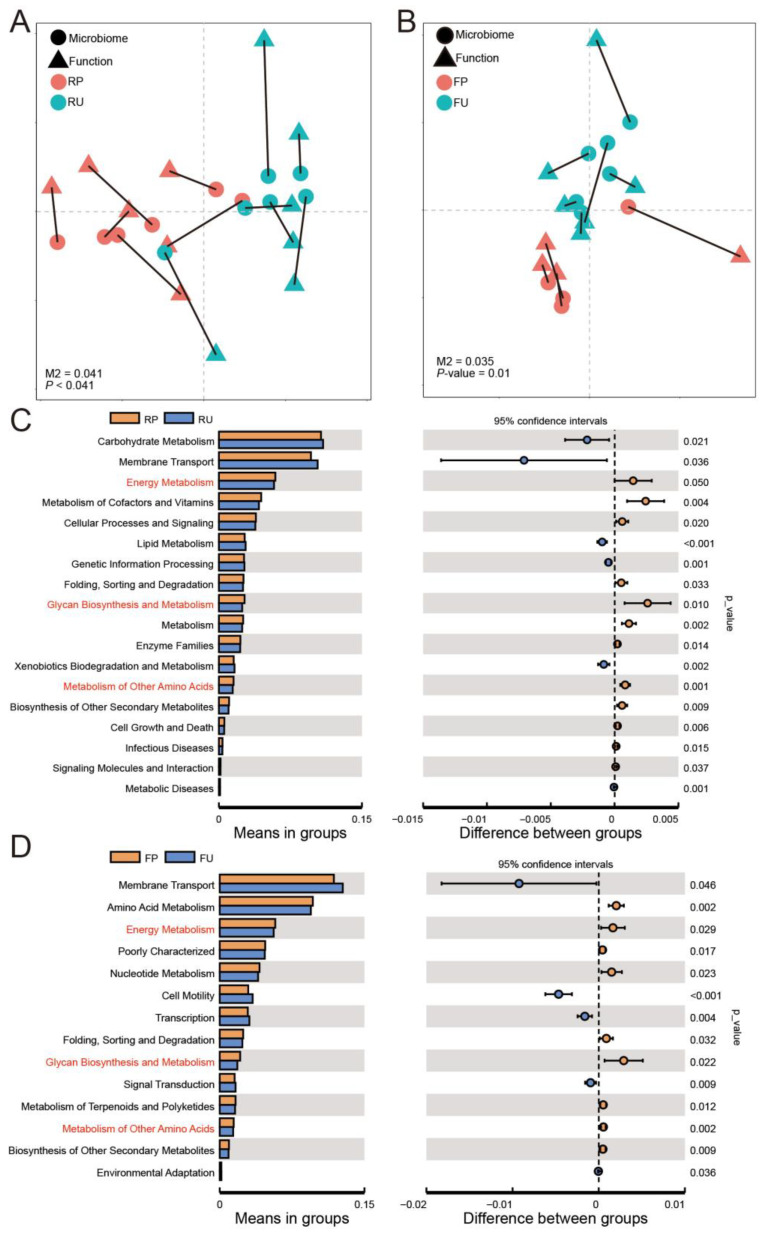
The analysis of microbial function. Procrustes analysis of the association between the microbiota and function in the rumen (**A**) and feces (**B**), respectively. The residual, M2, and *p*-value were generated by the ‘protest’ function, with *p* < 0.05 as the significance threshold. The significant differences in functions in the rumen (**C**) and feces (**D**), respectively. The functions marked in red were shared between the RP and FP groups. RP = rumen samples in the pasteurized colostrum feeding group; RU = rumen samples in the unpasteurized colostrum feeding group; FP = fecal samples in the pasteurized colostrum feeding group; FU = fecal samples in the unpasteurized colostrum feeding group.

Our investigation yielded significant differences in microbial functions between the groups receiving pasteurized and unpasteurized colostrum. The functional profiles of the rumen content and feces samples were found to cluster distinctly, as demonstrated by the Principal Component Analysis (PCA) (Figure S3). Furthermore, Procrustes analysis revealed a strong agreement between the microbial community structure and functional composition, both in the rumen (M2 = 0.04, *p* < 0.01, Figure 5A) and feces (M2 = 0.04, *p* = 0.01, Figure 5B). Specifically, in the rumen microbiota, the RP group exhibited a higher abundance of pathways associated with energy metabolism, metabolism of cofactors and vitamins, glycan biosynthesis and metabolism, and enzyme families, in comparison to the RU group (*p* < 0.05, Figure 5C). Conversely, pathways related to carbohydrate metabolism, lipid metabolism, and xenobiotic biodegradation and metabolism displayed lower abundances in the RP group (*p* < 0.05). In the fecal microbiota, the FP group exhibited higher abundances in pathways related to amino acid metabolism, energy metabolism, and glycan biosynthesis and metabolism, while pathways associated with membrane transport, cell motility, and signal transduction had lower abundances compared to the FU group (*p* < 0.05, Figure 5D).

## 4. Discussion

In our study, we observed that calves fed pasteurized colostrum exhibited significantly greater overall body weight and preweaning ADG [31]. This finding is consistent with one previous study, which also demonstrated that pre-weaned calves fed pasteurized colostrum experienced higher ADG compared to those fed unpasteurized colostrum [32]. Moreover, the impact of feeding pasteurized colostrum on the growth parameters of calves seems to have long-lasting effects. Armengol and Fraile [33] found that feeding pasteurized colostrum resulted in greater body weight of dairy heifers at their first calving, which aligns with the results of our study. Furthermore, additional studies have reported that feeding pasteurized colostrum has other notable benefits for pre-weaned calves. Godden et al. [18] and Armengol and Fraile [34] demonstrated that feeding pasteurized colostrum reduced the prevalence of pneumonia, diarrhea, and mortality rate in calves. These factors contribute to the overall improved growth performance of dairy calves fed pasteurized colostrum. However, in our study, the pasteurization of colostrum did not show a significant impact on diarrhea-related parameters or the prevalence of other diseases. This suggests that the improvement in growth performance attributed to colostrum may involve alternative mechanisms beyond solely enhancing calf health. Considering that colostrum contains active biomarkers, we conducted further investigations to explore the effects of pasteurized colostrum on hematological parameters. The analysis of these parameters can provide insights into potential alterations in metabolic and immunological frameworks. By examining the hematological parameters, we aimed to understand how pasteurized colostrum may exert its influence on the calf’s overall physiological and immune functions, which could in turn contribute to its improved growth performance.

Regarding the blood profile analysis, including parameters such as WBC, RBC, and MCH, no significant differences were observed between the two groups. However, it is important to note that all measured values fell within the previously reported normal ranges [35,36]. Notably, calves fed unpasteurized colostrum displayed lower values of W-SCR and PDW, along with higher values of W-LCR and W-LCC. This observation suggests a potential bacterial infection in the body, as a decrease in neutrophils in the blood is indicative of such an infection [37]. A recent study demonstrated that periparturient cattle with negative energy balance (NEB) experience a reduction in glycogen levels, which can lead to dysfunction in polymorphonuclear neutrophils and a higher incidence of perinatal diseases [38]. Conversely, an increase in W-SCR and PDW signifies chronic inflammation in calves [39]. Furthermore, a study investigating enzootic bovine leukosis (EBL) found a high proportion of lymphocyte subsets in the spleen of affected cattle, indicating an intense inflammatory response in this organ [40]. Collectively, these findings suggest that feeding pasteurized colostrum may continuously enhance the calves’ immune system, thereby improving their ability to defend against infections.

To gain further insights into the impact of pasteurized colostrum on the ruminal and fecal microbiota, we conducted a comprehensive analysis using 16S sequencing. In the rumen contents, feeding pasteurized colostrum did not significantly alter the microbial richness and diversity, although distinct microbial community clustering was observed between the groups receiving RP and RU. However, in the fecal samples, pasteurized colostrum feeding resulted in increased richness and diversity of the microbiota, leading to the formation of a distinct microbial community. Previous research indicated that calves fed pasteurized colostrum exhibited an increasing trend in hindgut bacterial diversity compared to those fed unpasteurized colostrum within six hours of birth [20]. However, few studies have explored the long-term effects of early feeding practices on gut microbiota and subsequent performance. Based on our findings and those of others, we speculate that the differences in microbial diversity resulting from the heat treatment of colostrum may persist into 180 d of age. Furthermore, our analysis revealed that the dissimilarities between the rumen content and fecal microbiota persisted until d 180, with each region harboring a highly specific microbiota structure, as indicated by Procrustes analysis. This distinction could be partially attributed to the physicochemical properties, such as pH, physiological function, and internal environment, which may contribute to the observed differences [29,41].

A comprehensive analysis of the bacterial composition at the genus level was conducted across all samples, leading to the identification of signature bacteria within each group. In both the RP and RU groups, the main genera detected were identified as *unidentified_Prevotellaceae* and *unidentified_Ruminococcaceae*. Notably, LEfSe analysis identified *unidentified_Prevotellaceae*, *Bacteroides* and *Lactobacillus* as the signature bacterial taxa with significantly higher abundance in the RP group. *Prevotellaceae*, in addition to its role as a primary fiber decomposer, has been found to exhibit a strong association with VFAs and the development of the rumen [42], which might account for the better growth performance observed in the calves of the RP group. The genus *Bacteroides* exhibits efficient degradation capabilities for cellulose and soluble saccharides [43]. *Lactobacillus* excels in utilizing non-structural carbohydrates to produce lactic acid and ethanol. A recent in vitro study demonstrated that *Lactobacillus* could inhibit the expression of inflammatory cytokines (such as IL-6, IL-1β, and TNF-α), thereby preventing mastitis [44]. In contrast, several pathogenic bacteria were identified as bacterial signatures in the RU group including *Allisonella*, *Atopobium*, *Corynebacterium* and *Lautropia*. *Allisonella* is known to produce histamine, and the accumulation of large amounts of histamine in the gastrointestinal tract can lead to acute inflammation associated with herbivores, such as spondylitis [45,46]. *Atopobium*, a hydrogen sulfide (H2S) producer, has been positively correlated with chronic gastrointestinal inflammation [47] and has been linked to genital tract infections [48]. *Corynebacterium,* recently identified as a zoonotic pathogen, poses concerns as it can spread from infected cattle to humans, causing respiratory diseases [49]. *Lautropia*, on the other hand, may serve as a biomarker to predict pro-inflammatory responses in tuberculosis cases [48]. Consequently, animals in the RU group exhibited the lowest ADG and increased sensitivity to inflammatory markers. Although pathogenic bacteria may competitively colonize the gut, pasteurization effectively reduces the abundance of pathogenic bacteria and allows the dominance of some probiotic bacteria including *Lactobacillus*, *Bifidobacterium*, *Bacteroides*, *Faecalibacterium* and *Prevotella* [50,51,52]. In this study, we also observed the presence of some probiotic bacteria in the gut of calves fed pasteurized colostrum including *Alloprevotella* and *Parabacteroides*. Both bacteria are capable of producing short-chain fatty acids (SCFAs). Additionally, they contribute to the maintenance of gut microecological balance, reduce the production of pro-inflammatory cytokines by gut cells, and enhance gut integrity [53,54]. Furthermore, metabolites derived from certain probiotics can act as ligands that specifically bind to receptors of relevant signaling pathways, thereby promoting gut development and maintaining homeostasis in the host. For instance, SCFAs produced by acid-producing bacteria serve as ligands for G-protein-coupled receptors (GPCRs), activating the GPR41/43 pathway to modulate epithelial cell turnover and stimulate gut epithelial proliferation [55]. Meanwhile, butyrate suppresses the HDAC8/NF-κB signaling pathway, leading to the upregulation of SLC26A3 and key tight junction proteins, thereby enhancing gut epithelial integrity and barrier function [56].

In addition, our results indicate that although the microbiota composition in different segments of the gut may differ, certain specific bacteria consistently appeared in both the rumen and feces, suggesting a possible longitudinal spread along the gastrointestinal tract. *Alloprevotella*, *Parabacteroides*, and *unidentified_Prevotellaceae* were the most shared bacterial signatures. In our previous study, we noted that microbiota present in colostrum represent an important source of gastrointestinal microbiota for newborn calves [57]. Combined with results of this experiment, we speculated that some early-colonizing microbes derived from colostrum may persist stably in the calf digestive tract. Furthermore, pasteurization eliminates most pathogenic bacteria, thereby providing probiotic bacteria with a competitive advantage in ecological niche competition within the complex microbial community, facilitating more rapid proliferation.

The sparCC algorithm was employed to identify the interactions between the microbiome and specific characteristics, namely VFAs and blood routine parameters. VFAs serve as the primary end-products resulting from the microbial fermentation of solid feed, supplying essential energy for the development of the rumen and intestines [58]. Although pasteurization treatment did not exhibit a significant direct impact on VFA concentrations in either the rumen or feces, we consistently observed notable associations between bacteria, including distinct signatures within each group, and VFAs. This suggests that it remains feasible to modulate VFAs by artificially manipulating the gut-related microbiota [59]. Furthermore, we investigated the correlation between blood immunity and microbiota indices. A growing amount of evidence suggests that microbial communities play a pivotal role in host immunity [60,61] through intricate crosstalk involving the microbiota, metabolites, microbial components, and host immune cells [62]. To gain comprehensive insights into the potential connections between the microbiota and the host, future studies could employ advanced techniques like metabolomics. By employing such methodologies, researchers can further elucidate the intricate relationships between microbial communities, metabolites, and the host’s immune system, thus deepening our understanding of host–microbiota interactions.

At the functional level, a notable association between microbiota and their functionality in both the rumen and feces implies that a comparable microbial community leads to a consistent functional structure. Irrespective of whether it is observed in the rumen or feces, calves fed pasteurized colostrum exhibit a greater degree of shared active metabolism involving energy, amino acids, and glycans in comparison to those fed unpasteurized colostrum. It is worth noting that specific microbiota fulfill distinct functions, thereby implying that discrepancies in function typically arise from corresponding variations in microbiota composition [22]. Consequently, the alteration of microbial composition resulting from the consumption of pasteurized colostrum may play a pivotal role in determining these functional disparities.

**Figure 6:**
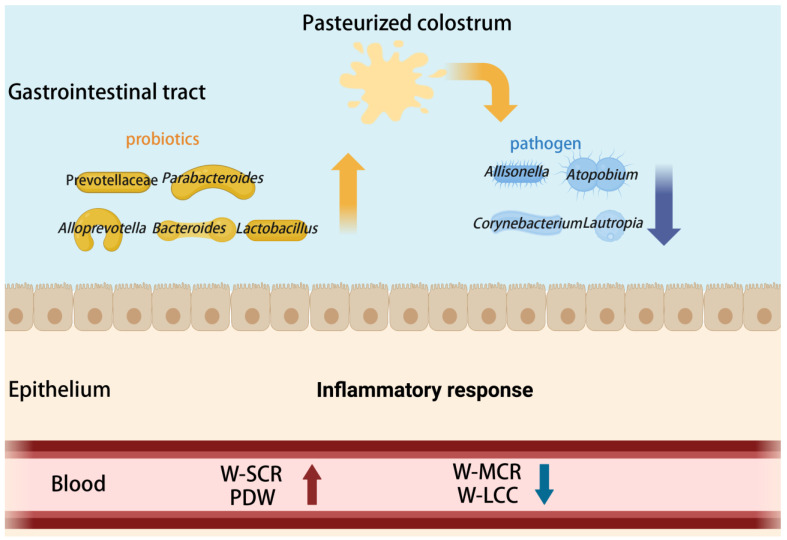
A comprehensive response of gut microbiota to pasteurized colostrum. W-SCR = lymphocyte ratio; PDW = platelet distribution width; W-MCR = lymphocyte ratio; W-LCC = lymphocyte ratio.

Overall, we propose that pasteurization of colostrum effectively inhibits the proliferation of pathogens in both the foregut and hindgut of calves, increases the abundance of potential probiotics, and thereby improves host growth performance via microbial metabolism, while also enhancing blood immune competence and reducing inflammatory responses (Figure 6).

## 5. Conclusions

This study demonstrated the positive effects of pasteurized colostrum on the growth, blood immunity, and gastrointestinal microbiota of calves. Pasteurized colostrum feeding in the initial stages of life significantly improved final body weight and preweaning ADG and alleviated inflammatory responses of calves until d 180 of age. These positive effects may be due to pasteurized colostrum enhancing the abundance of beneficial bacteria and associated functions in both the rumen and hindgut. This study highlights the crucial interplay between microbiota and host blood immunity and performance. However, this study still has several limitations. For instance, we only investigated the gastrointestinal microbiota of calves at the end of the experiment and did not perform continuous measurements at multiple time points to assess its temporal dynamics. Similarly, growth performance was not evaluated with more frequent and longitudinal measurements to track the long-term developmental effects of pasteurized colostrum. Furthermore, although correlation analysis revealed potential associations between certain microbiota and host metabolic parameters, further mechanistic validation is still required. These issues will be addressed in our future studies.

## Figures and Tables

**Table 1:** Effects of feeding pasteurized colostrum on body weight and withers height of dairy calves.

	Treatment ^1^			*p*-Value ^2^		
Items	PC	UC	SEM ^2^	T	t	T × t
Calves, *n*	16	16	—	—	—	—
BW ^3^, kg						
Initial	38.6	41.1	0.81	0.13	—	—
30 d	71.3	66.3	1.33	0.06	—	—
60 d	110.1	108.1	1.43	0.47	—	—
120 d	163.3 ^a^	155.2 ^b^	1.92	0.03	—	—
150 d	200.1	191.1	2.54	0.08	—	—
180 d	228.9	225.4	4.53	0.55	—	—
Overall	135.4	131.3	3.43	<0.01	<0.01	0.26
ADG ^4^, g/d						
Preweaning	1021.0 ^a^	950.9 ^b^	16.86	0.04	—	—
Postweaning	1080.0	1073.0	21.37	0.76	—	—
Overall	1050.5	1029.0	14.24	0.12	<0.01	0.30

^1^ PC = pasteurized colostrum feeding group; UC = unpasteurized colostrum feeding group. ^2^ T = treatment, t = time, T × t = the interaction between treatment and time. ^3^ BW = body weight. ^4^ ADG = average daily gain. ^2^ SEM = standard Error of the Mean. a,b Values in a row with no common superscripts differ significantly (*p* < 0.05).

**Table 2:** Effects of feeding pasteurized colostrum on the diarrhea of dairy calves in the preweaning period.

Items	Treatment ^1^		SEM ^2^	*p*-Value
	PC	UC		
Calves, *n*	16	16		
Diarrhea days, d	11.16	12.58	0.480	0.14
Diarrhea frequency, %	7.48	7.59	0.107	0.63

^1^ PC = pasteurized colostrum feeding group; UC = unpasteurized colostrum feeding group. ^2^ SEM = standard Error of the Mean.

**Table 3:** Effects of pasteurized colostrum on the hematological profile of dairy calves at d 180.

Items ^2^	Treatment ^1^		SEM ^3^	*p*-Value
	PC	UC		
Calves, *n*	16	16		
WBC, 10^9^/L	11.16	12.58	0.480	0.14
RBC, 10^12^/L	7.48	7.59	0.107	0.63
HGB, g/L	101.00	103.14	1.999	0.60
HCT, %	32.14	32.50	0.337	0.61
MCV, fL	42.40	43.49	0.318	0.09
MCH, pg	12.93	13.19	0.264	0.64
MCHC, g/L	330.61	335.90	5.237	0.62
PLT, 109/L	401.21	402.43	10.843	0.96
W-SCR, %	56.89	53.46	0.603	<0.01
W-MCR, %	10.54	10.54	0.241	0.99
W-LCR, %	32.56	36.00	0.611	<0.01
W-SCC, 10^9^/L	6.34	6.75	0.265	0.45
W-MCC, 10^9^/L	1.19	1.31	0.050	0.23
W-LCC, 10^9^/L	3.61	4.51	0.197	0.02
PDW, %	10.30	9.29	0.220	0.02
MPV, fL	7.79	7.48	0.082	0.06
RDW-SD, %	20.01	21.14	0.366	0.87
RDW-CV	0.16	0.15	0.042	0.08
P-LCR, %	6.83	6.89	0.097	0.75

^1^ PC = pasteurized colostrum feeding group; UC = unpasteurized colostrum feeding group. ^2^ WBC = total leukocyte count; RBC = number of red blood cells; HGB = hemoglobin; HCT = hematocrit; MCV = mean corpuscular volume; MCH = mean corpuscular hemoglobin; MCHC = mean corpuscular hemoglobin concentration; PLT = platelet; W-SCR = lymphocyte ratio; W-MCR = monocyte ratio; W-LCR = neutrophil ratio; W-SCC = total lymphocyte count; W-MCC = monocyte count; W-LCC = neutrophil count, PDW = platelet distribution width; MPV = mean platelet volume; RDW-SD = red cell volume distribution width; RDW-CV = red blood cell distribution width; P-LCR = large platelet ratio. ^3^ SEM = standard Error of the Mean.

**Table 4:** Effects of feeding pasteurized colostrum on rumen and fecal pH and VFAs of dairy calves at d 180.

Items	Treatment ^1^		SEM ^2^	*p*-Value
	PC	UC		
Calves, *n*	6	6		
Rumen fermentation				
pH	5.85	5.95	0.030	0.33
Acetate, mmol/L	64.50	62.80	4.027	0.84
Propionate, mmol/L	34.20	36.40	1.475	0.50
Isobutyrate, mmol/L	1.11	1.09	0.132	0.57
Butyrate, mmol/L	17.80	17.80	1.320	0.99
Isovalerate, mmol/L	1.40	1.36	0.238	0.51
Valerate, mmol/L	2.90	2.97	0.265	0.30
Total VFA ^2^, mmol/L	121.90	129.00	7.760	0.47
Fecal fermentation				
pH	5.70	5.71	0.030	0.84
Acetate, mmol/L	62.20	70.50	6.190	0.75
Propionate, mmol/L	14.90	15.50	1.452	0.89
Isobutyrate, mmol/L	1.78	1.77	0.233	0.83
Butyrate, mmol/L	5.17	5.71	0.620	0.51
Isovalerate, mmol/L	1.83	1.44	0.301	0.76
Valerate, mmol/L	1.75	2.30	0.372	0.51
Total VFA, mmol/L	87.60	97.90	7.890	0.78

^1^ PC = pasteurized colostrum feeding group; UC = unpasteurized colostrum feeding group. ^2^ VFA = volatile fatty acid. ^2^ SEM = standard Error of the Mean.

## Data Availability

The datasets used and/or analyzed during the current study are available from the corresponding author on reasonable request. The sequencing data in this study is available via the NCBI Sequence Read Archive using accession number BioProject PRJNA817406.

## Supplementary Materials

The following supporting information can be downloaded at https://www.mdpi.com/article/10.3390/microorganisms13092089/s1: Table S1. Nutritional composition and immune factor level of colostrum. Table S2. Milk feeding regime. Table S3. Nutritional components of calf starter. Figure S1. The dry matter intake (DMI) of starter of calves during the preweaning period (from d 0 to d 70) in the PC and UC groups. Figure S2. Microbial composition at the phylum level in response to pasteurized colostrum feeding. Figure S3. Functional diversities and structure of the rumen and feces in calves.
