# Supplementary material for: Pasteurized Colostrum Improves Blood Immunity and Gastrointestinal Microbiota in Dairy Calves from Birth to 180 Days of Age
Source: Microorganisms. 2025 Sep 8;13(9):2089. doi: 10.3390/microorganisms13092089 (PMC12472721; doi:10.3390/microorganisms13092089)
Supplement: Supplementary file 1 [file microorganisms-13-02089-s001.zip › microorganisms-3797250-supplementary.docx]

**Table S1.** Nutritional composition and immune factor level of colostrum

| **Items** | **Treatment** | |
| --- | --- | --- |
|  | **PC** | **UC** |
| Nutrients |  |  |
| Fat, g/100g | 6.37 | 8.86 |
| Protein, mg/mL | 0.67 | 0.99 |
| Lactose, mg/mL | 0.54 | 0.63 |
| Immune factor |  |  |
| IgA, mg/mL | 3.44 | 3.94 |
| IgG, mg/mL | 42.52 | 47.26 |
| IgM, mg/mL | 2.26 | 2.68 |

**Table S2.** Milk feeding regime

| Day | Feeding quantity, L |
| --- | --- |
| 2 d | 5.0 |
| 3-6 d | 8.0 |
| 7-50 d | 10.0 |
| 51 d | 10.5 |
| 52 d | 9.0 |
| 53 d | 7.5 |
| 54-55 d | 6.0 |
| 56-57 d | 4.5 |
| 58-59 d | 3.0 |
| 60 d | 1.5 |
| Total period | 581.5 |

**Table S3.** Nutritional components of calf starter

| **Items** | **Calf starter^1^** | **Oat hay** |
| --- | --- | --- |
| Chemical composition, % of DM basis | | |
| DM | 88.40 | 92.39 |
| CP | 23.98 | 7.28 |
| EE | 2.51 | 1.97 |
| NDF | 20.40 | 46.25 |
| ADF | 9.65 | 25.30 |
| Ash | 7.43 | 8.12 |
| NFC | 45.68 | 36.38 |
| Ca | 1.04 | - |
| P | 0.42 | - |

DM: Dry matter; CP: Crude protein; EE: Ether extract; NDF: Neutral detergent fiber; ADF: Acid detergent fiber; Ash: Crude ash; NFC = 100 – (NDF + CP + EE + Ash).

^1^Calf starter was supplied by Modern Farming Co. Ltd., and contained 33.6% corn, 18.8% fermented soybean meal, 14.1% soy hulls, 10.5% soybean meal, 6.3% wheat bran, 6.3% wheat flour, 5.4% whey powder, 5% premix compound (contained vitamin A 200,000 IU/kg, vitamin D 25,000 IU/kg, vitamin E 2,000 IU/kg, manganese 0.6 g/kg, iron 0.4 g/ kg, copper 0.5 g/kg, zinc 2g/kg, cobalt 4 mg/kg, iodine 16 mg/kg, and selenium 4 mg/kg.) on a DM basis.


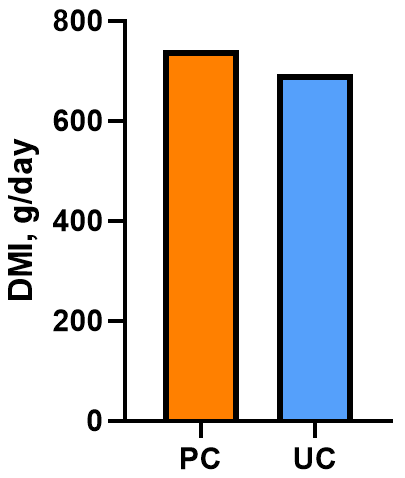


**Figure S1.** The dry matter intake (DMI) of starter of calves during the preweaning period (From d 0 to d 70) in the PC and UC group. PC = pasteurized colostrum feeding group; UC = unpasteurized colostrum feeding group. Since the preweaning calves were kept together in the pens of each group respectively instead of each calf housing in the outdoor plastic individual hutches. We could only calculate the average DMI of each group of calf. After the calves were weaned, all the calves in both groups were transferred to the heifer barn until the completion of the study. Hence we did not record and compare the DMI of calves during the postweaning period between these two groups.

**
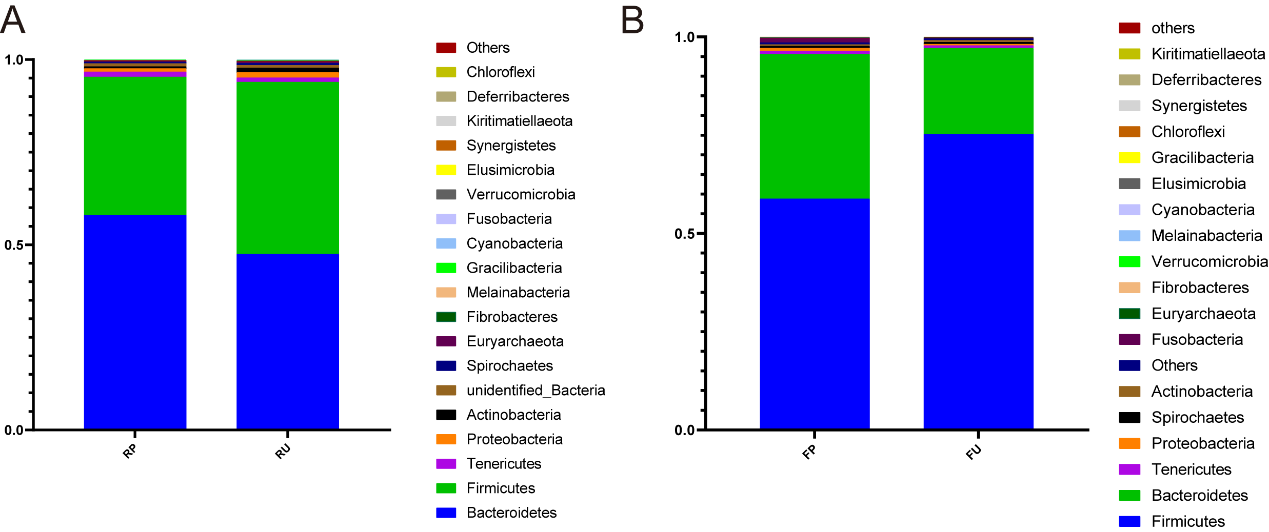
**

**Figure S2.** Microbial composition at the phyla level in response to pasteurized colostrum Feeding. Stacked bar charts demonstrate the abundance of the top 20 common phylum in the rumen (**A**) and feces (**B**) of dairy calves respectively.

**
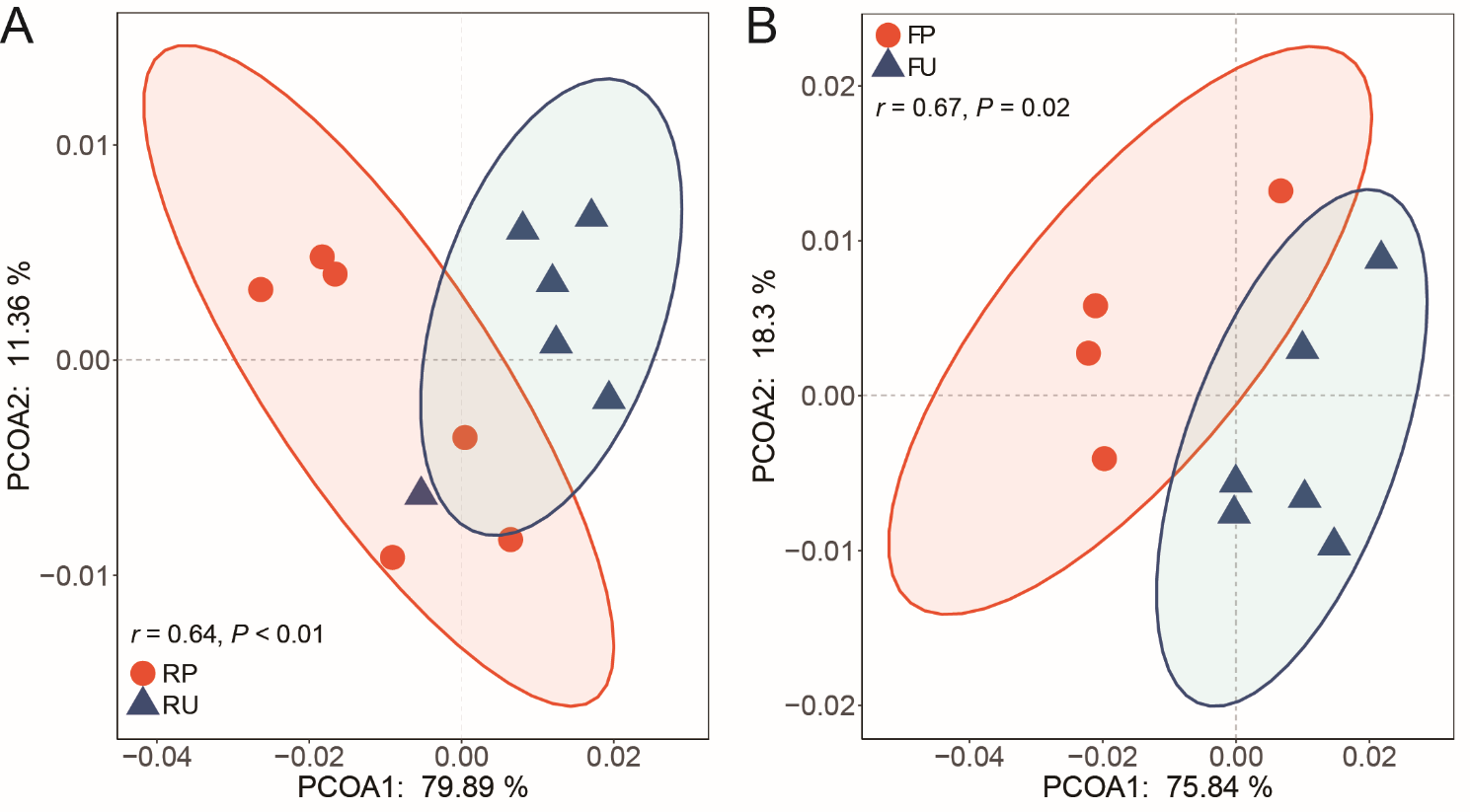
**

**Figure S3.** Functional diversities and structure of the rumen and feces in calves. **A, B** The principal coordinate analysis (PCoA) based on the Bray–Curtis with ANOSIM analysis. Each point represents a unique sample. Different colors represent different groups. RP = rumen samples in the pasteurized colostrum feeding group; RU = rumen samples in the unpasteurized colostrum feeding group; FP = fecal samples in the pasteurized colostrum feeding group; FU = fecal samples in the unpasteurized colostrum feeding group.
